# Cancer, COVID-19, and the need for critique

**DOI:** 10.12688/wellcomeopenres.16404.2

**Published:** 2021-06-11

**Authors:** Cinzia Greco, Ignacia Arteaga, Clara Fabian-Therond, Henry Llewellyn, Julia Swallow, William Viney

**Affiliations:** 1 Centre for the History of Science Technology and Medicine (CHSTM), University of Manchester, Manchester, M13 9PL, UK; 2 Department of Social Anthropology, University of Cambridge, Cambridge, CB2 3RF, UK; 3 Department of Anthropology, University College London, London, WC1H 0BW, UK; 4 UCL Division of Psychiatry, University College London, London, W1T 7NF, UK; 5 Usher Institute of Population Health Sciences and Informatics, University of Edinburgh, Edinburgh, EH8 9LD, UK; 6 Department of Anthropology, Goldsmiths, University of London, London, SE14 6NW, UK

**Keywords:** Cancer, COVID-19, Social Sciences, United Kingdom

## Abstract

In this open letter we examine the implications of the coronavirus disease 2019 (COVID-19) pandemic for cancer research and care from the point of view of the social studies of science, technology, and medicine. We discuss how the pandemic has disrupted several aspects of cancer care, underscoring the fragmentation of institutional arrangements, the malleable priorities in cancer research, and the changing promises of therapeutic innovation. We argue for the critical relevance of qualitative social sciences in cancer research during the pandemic despite the difficulties of immersive kinds of fieldwork. Social science research can help understand the ongoing, situated and lived impact of the pandemic, as well as fully underline its socially stratified consequences. We outline the risk that limiting and prioritising research activities according to their immediate clinical outcomes might have in the relational and longitudinal understanding of cancer practices in the UK. Finally, we alert against potential distortions that a “covidization” of cancer research might entail, arguing for the need to maintain a critical point of view on the pandemic.

## Disclaimer

The views expressed in this article are those of the author(s). Publication in Wellcome Open Research does not imply endorsement by Wellcome.

## The entanglements between cancer care and COVID-19: A multi-layered disruption

The coronavirus disease 2019 (COVID-19) pandemic has abruptly altered the lives of millions and caused major disruptions to those classified as the most vulnerable segments of the population by the UK Government, including patients living with cancer. Millions of elective operations have been postponed ( Sample, 2020), with pathways altered (see Cancer Research UK news article), waiting times increased, screening programs suspended ( Maringe *et al.,* 2020). Medical personnel have also been placed under significant and sustained stress (see BMA mental health wellbeing report). There has also been a significant upheaval in the ordinary activities of NHS trusts, professional bodies and structures already enduring austerity and managerialisation policies. Such upheaval had marked consequences for cancer care. Since the start of the pandemic in the UK, several articles have brought forward the testimonies of patients whose treatments have been interrupted or delayed or who were alerted that they would have not found a place in ICU had they contracted COVID-19 ( Hughes, 2020).

Modelling approaches to statistically estimate the disruption brought by the pandemic are focusing mainly on excess mortality and life-years lost, something that it is in itself difficult to ascertain within the current time-frame. Sud *et al.* (2020), focusing on mortality derived from diagnostic delays, alert that “[u]nlike acute pathologies, such as stroke and myocardial infarction, the true excess mortality due to COVID-19-related disruption to cancer pathways will not be fully evident for 10 years or longer”. These distal statistical approaches make it difficult to garner a full understanding of how and for whom cancer services have been disrupted, particularly because such modelling work claims that we will only be able to understand the disruption after the pandemic, when mortality data becomes available and clarified. This risks occluding the real-time, on the ground, disruptions to cancer services. We therefore require different empirical and theoretical approaches to document and understand these developments.

Longitudinal observational work within the social sciences is primed and critical to illuminating the multiple tensions that are informing clinical dynamics. Ethnographic work, in particular, privileges in-depth accounts of the “on the ground realities” experienced by multiple stakeholders. It offers unique insights into different timescales, experiences, and points of view, and further how these are related and held in tension. Yet, such work in cancer contexts has also been interrupted during the pandemic, due to a number of conditions and for a variety of reasons. Patients, relatives and healthcare professionals who experience proximal disruption are required to deal with novel situations and improvise care-work practices within changing terrains. Yet, disruptions are emerging across the cancer care continuum and are not limited to pausing screening and diagnostic delays, but also involve patients already in treatment and those in follow-up care. Those who are affected by advanced cancer often rely on clinical trials to access potentially life-lengthening treatments. Because of their bodily vulnerability, this segment of patients is particularly impacted by the suspension of research on cancer and of clinical trials. However, we must keep in mind that some of the features that are considered to be disruptions for the patients might not be perceived as such by the medical professionals, and vice-versa. The difficulty of in-person consultations, in some cases substituted by telephone and/or online exchanges, as well as not being able to have an accompanying person (e.g. friend/relative) attend appointments, have a different impact for patients than for clinical professionals – these are consequences that are often difficult to anticipate. Understanding the different ways in which cancer care has been impacted by the pandemic therefore requires to look at an ample range of alterations, both immediate and long-term, to cancer care. Such an understanding – a project for which the social sciences are pivotal – is greatly needed in order to plan supporting mechanisms for the patients and the full reactivation of services.

This open letter is the result of an online workshop organised in late September 2020 on the mutual implications between cancer care and COVID-19, involving six researchers whose scholarship concerns the practices, experiences and ideologies informing the fields of cancer in the UK. The workshop was motivated by the need to make sense of the barriers we must navigate while carrying out cancer ethnographies in the UK. It extended from there to reflect on the impact of COVID-19 on cancer care, including some aspects of cancer care that remain uncertain because of the lack of ethnographic or other kinds of data.

## The fragmentation of cancer care

In addition to the disruption to NHS services, charities working both on cancer care and cancer research have experienced significant difficulties since the beginning of the pandemic, with income yielded from donations dropping suddenly and ensuing restructurations of their own operations (see Cancer Research UK blog on funding cuts). While the UK has been characterised historically by the prominent role of the state in funding and providing healthcare, with a somewhat limited role for “for-profit” or private social actors, since the 1980s there has been an increase in the private healthcare sector and its partnerships with formerly public research and clinical institutions. These partnerships become visible through particular mechanisms, such as in the increasing outsourcing of NHS services to private firms, the growth of private research enterprises partnering with NHS bodies to harness rich and centralised data and to invest in translational research, and the role of private charities in enabling research and delivery of cancer therapies. Medical charities in the UK not only fund basic and population-based cancer research, but they also offer guidance and support to patients with cancer both outside and inside the NHS, also delivering pan-cancer rehabilitation services across the country (e.g. Macmillan Cancer Support among others). The ongoing crisis that cancer charities are facing reveals a significant paradox. Most charitable organisations have developed either to cover areas that had limited public support (e.g. Marie Curie provides community palliative care) or to cover areas from which the public services have retreated, with hindsight, such as the case of psycho-social support.

Due to the pandemic, even a government guided by a political party historically hostile to public spending has taken some exceptional policies to alleviate bottlenecks and improve access to treatments in a healthcare service overwhelmed by demand. This has shown the possibility, and arguably the necessity, for centralising healthcare services and resources under public control to deal both with peaks in the number of cases, and the continued provision of ordinary healthcare needs. However, a significant part of the care that patients rely on is managed through charities, which because of their financing model, legal status, and organisational structure, are difficult to centralise under public control. The risk is that even if the government decides to allocate the resources necessary to avoid major disruptions to cancer services during a public health crisis, an important part of the current offer of cancer care in the UK might be significantly affected.

COVID-19 is revealing the fragmentation in cancer care that is located at the intersection between private and public management, charity and industry, also evidencing the persistent effect of austerity policies in the healthcare and social care domains. In this fragmented landscape, some patients find comfort and support in less institutionalised contexts, such as neighbourhood, friendship or cancer support groups. However, the requirement for social distancing during the pandemic has also altered the availability of these forms of help, thus worsening the quality of life of cancer patients who have needed to shield to minimize the risk of contracting the virus suffering from already compromised immune-defence mechanisms.

In this context, it is urgent that we study the real-time changes materialising prospectively so we can both shed light on their implications for all those affected (see Vindrola Padros *et al.,* 2020) as well as develop rich and sustained scholarship throughout the pandemic. Such analysis better shows how forces and structures which might appear intractable were not always inevitable, as much long-term ethnographic work attests ( Biehl & Locke, 2017).

## (Re)shaping research practices during COVID-19: Challenges and possibilities

Social studies of science, technology and medicine continue to play a vital role in understanding interactions between biology, subjects, and environments. Eschewing simple and reductive accounts, they have tackled social worlds in their complexity and considered the mutual constitution of these domains and entities. Recent scholarship has explored how novel understandings of biology reshape our worlds, organise our societies, and inform how we understand ourselves ( Wahlberg, 2018). Other contributions have mapped the social effects of biomedicine, informing changing perceptions of the body, normative definitions of what is considered to be a desirable behaviour, and public entitlements to health and care ( Lock & Nguyen, 2011; Petryna, 2002). Moreover, many social science scholars have delved into the lived experiences of those seeking care, unpacking the particular relationships through which people affected by conditions withstand disease-related suffering and sometimes find relief ( Das, 2015). This scholarship has also developed critical lenses on emergence, provisionality, revolutionary change and stabilisation in science, technology and care ( Keating & Cambrosio, 2003). In the field of oncology, social science disciplines have played and continue to have important roles in understanding relations among experts and publics, perceptions of risk, experiences of the cancerous body, inequalities in access to standard and experimental treatments, professional practices, informal care work, and the involvement of diverse publics in cancer-related research ( Arteaga, 2019; Arteaga Pérez, 2020; Day *et al.*, 2016; Greco, 2016; Greco, 2019; Kerr & Cunningham-Burley, 2015; Kerr *et al.*, 2018; Llewellyn *et al.*, 2018; Llewellyn & Higgs, 2020; Swallow *et al.*, 2020; Therond *et al.*, 2020). Through an array of ethnographic research practices including interviews, observation, participatory techniques, and archival research, social sciences scholarship has contributed to our understandings of the promises, ambivalences, complexities and difficulties within the biomedical field from the perspective of different stakeholders, including patients, caregivers, medical professionals, scientists, and interested publics.

Ethnographic methods can make an important contribution to how the COVID-19 pandemic has been understood and managed. First, it can understand arising and unfolding issues. To mention two examples, the lived realities of those affected by COVID-19 will be lifelong. Enduring social and health inequalities has made COVID-19 different for different people, as the Covid Realities project has shown. Moreover, the response of the UK government has been criticised for its temporary over-reliance on behavioural experts and its support of individualised preventive measures. These approaches, we argue, have ignored equally significant collective measures and responses that involve the re-organisation of working life and the economy. By describing “people in context” ethnographic approaches can combine state-sanctioned individual responses with collective and other perspectives whose conception of personhood is not reducible to pre-given national, ethnical, biomedical, or other categories. Second, ethnographic work is crucial to give a more situated and therefore relational understanding of the experiences and mechanisms through which care is negotiated and embodied. An essential part of what matters to people cannot be captured by quantitative metrics. Or by numbering practices underpinned by methodological individualism. Ethnographic analysis can provide rapid and systematic estimation of social challenges that affect communities’ resilience and their capacity to withstand the effects of the pandemic. Reports such as A Right To Care, for example, offer important insight into how we can make recovery plans and policies workable and inclusive.

Over recent months each of us - as social scientists, members of families, and civic actors - have found ourselves dislocated from our assumptions and routines pre-COVID-19. This visceral sense of dislocation contributes significant social and professional challenges. However, it also provides a new critical space to reflect, amongst other things, on how we are able to conduct and communicate our research in this time of COVID-19.

As large portions of our social life have been redirected online, the social sciences are also in the process of adapting methods and theoretical frameworks to better understand the challenges posed by the pandemic to those affected by cancer either personally or professionally. The discussion emerging from the workshop demonstrated how, as social scientists, our current research practices have not only been constrained by the pandemic but also reshaped. As ethnographers, we are all too aware that barriers to the “on-the-ground,” in person, physically-situated component of our work – momentarily or for longer periods – poses risks to research practice. Online and remote research, while offering safer access to the communities with whom we work, cannot fully grasp the material and contextual nuances that inform people’s everyday efforts; it changes the relational dynamics between the ethnographer and participant, and the opportunities for participant-observation in field sites as a core ethnographic endeavour.

Part of the difficulties we are experiencing in relation to the current constraints of immersive and situated research methods in social research parallel those of medical professionals and patients, whose own relations have also been redrawn. For medical professionals, virtual approaches bring challenges to usual techniques of assessing, supporting, responding to and caring for patients. For patients, daily routines have been radically disrupted, care provision has been redefined with cancelled appointments and procedures, and virtual follow-up clinics. All are subject to increased uncertainties in their lives and work, coupled with on-going emotional labour that characterises provisional and highly precarious knowledge, policy and practice about the pandemic.

Moreover, online recruitment strategies in social science research can privilege participants who tend to have more resources and time at their disposal, inevitably skewing the range of lived realities we are able to encounter as researchers, given that internet access reflects wider social and structural inequalities. As with social scientists, clinical research coordinators must grapple with the sample bias that the COVID-19 pandemic might pose, considering their own mandates to protect patients from the intrusion of research into clinical pathways, which might stand for an essential component of care. Whilst clinical experiences and practices are changing, our methodologies start reflecting and recording those changes.

This pandemic, while sudden and harsh, is not the cause of all the current problems in healthcare settings, but a contributor, catalyst and powerful amplifier. The pandemic has exacerbated existing fragilities and reactivated old fault-lines that research on healthcare services and oncology has already shown ( Arteaga *et al.*, 2019). With this in mind, we think it is important to resist the temptation to divide the reality in a pre- and post-COVID-19 and instead look to critically reflect on the role of social science and qualitative research specifically for addressing these challenges whilst also reflecting on the emergence of alternative knowledge-making practices.

## The covidization of research: the role of qualitative social science research

Aware of the continuity between the clinical, financial and political difficulties already existing in the National Health Service before March 2020, we are cautious to not fall into the “covidization” of all scientific research ( Pai, 2020). By “covidization” we refer to the tendency of approaching the study of the COVID-19 pandemic, by attributing all the problems that have emerged to the pandemic itself. We argue that rationing available resources and directing them to this research agenda risks overlooking other enduring problems within the organisation of healthcare and medicine. With restrictions to social life still in place, the pandemic seems to be, if not the only, certainly the dominant interpretative framework to analyse the present, especially in the biomedical field. Embedded at various levels, from funding, research portfolio management, research ethics, study sponsorship, capacity and capability assessments; and through the discretionary decisions of clinical teams, this framework has had significant impacts on the development of research infrastructures and consequently the kinds of knowledge that are produced. A recent consensus-building paper produced by Cancer Core Europe ( van de Haar *et al.*, 2020) suggests to “reduce preclinical research activities to a bare minimum” and “stop patient inclusion for clinical studies or trials requiring additional actions and/or visits” (667) – this, in practice, makes translational cancer research an exception. Moreover, in a demonstration of “ethical variability” in clinical care ( Petryna, 2005), the authors argue for the need for the selective “adjustment” (that is, de-escalation) of anticancer therapeutic regimes during the pandemic. This involves the hypothesis that the de-escalated treatments could be normalised post-pandemic if the clinical outcomes are not inferior to those of the pre-pandemic treatments. As a prime example of the covidization of research, the published roadmap claims that “[t]he COVID-19 pandemic may offer a unique window of opportunity for retrospective trials, assessing the non-inferiority of de-escalated treatment regimens, which may be difficult to perform under normal conditions for ethical reasons” (670).

The redefinition of research activities during the pandemic intersects with and reinforces pre-existing professional, institutional, disciplinary and epistemological hierarchies. Whether pre-approved studies are funded by the National Institute of Health Research (NIHR; the largest funder of health and care research in the UK) or not, frameworks such as the NIHR Restart determine what is considered “urgent” and hence of essential value (defined as “patient benefit” and health delivery “cost-efficacy”). NIHR Restart then sifts through particular studies in relation to present and potential risks, prioritising certain (clinical, measurable) approaches while stalling others (social, critical) where value is difficult to estimate.

Although such logics of urgency and priority are inevitable and important in contexts of radically compromised capacity, we are keen to advocate for guiding principles that accommodate the kinds of critical social science work outlined above, which examines and bears witness to the social consequences of disease and care, and the ongoing articulation of ethical frameworks across a variegated society. In particular, we have in mind approaches that offer ethnographic approaches and those that reside beyond the limits of the clinic, which nevertheless offer an essential contribution to the understanding of the social impact and personal stakes that the pandemic has brought onto healthcare services and people’s everyday lives. If COVID-19 has surfaced the UK’s enduring health inequalities into public awareness, then the organisational streamlining and prioritisation of clinical and biomedical research within the NHS risks, paradoxically, sidelining the social scientific research that can bear witness to the wider social dynamics that are core to COVID-19’s uneven effects. At stake in this “covidization” of research is thus both a broader and more nuanced understanding of the pandemic and its effects and the displacement of important work in other arenas that are not explicitly deemed “COVID-related” ( Pai, 2020). Indeed, just as the pandemic exposes and is exacerbated by socio-cultural, economic and political disparities, it is critical that ethnographers are enabled to participate in the disentanglement of the on-the-ground effects of COVID-19 on cancer treatment and care practices across and beyond the UK.

## Looking at the future: Intersections between COVID-19 and the promise and practice of personalised cancer medicine

Important innovations in cancer treatments have been linked to the identification of specific biological markers, allowing for targeted therapies for specific subgroups of patients. It is these kind of stratification practices that are behind the promises of what is commonly called “personalised” medicine. The COVID-19 pandemic, and the subsequent success and rollout of the UK vaccination programme, has, in many ways, brought into sharp relief the “circulation of scientific promises” attached to the development and delivery of personalised medicine in oncology and rare diseases ( Sturdy, 2017:31).

Yet, we know that the impact of COVID-19 on cancer research communities is profound, as funding and public/private investment is squeezed, and human and non-human resources are re-routed to help tackle the virus’s effect on society and citizens (see Cancer Research UK Open Letter to researchers). We have seen how laboratory closures, as a result of the national lockdown, slowed down scientific progress thus exacerbating any mismatch between upstream promises in cancer research and their downstream translation into clinical care (see Cancer Research UK researchers lockdown experience survey). As *Genome UK: The Future of Healthcare* ( Department of Health and Social Care, 2020:19) outlined, some of the most exciting developments in early detection involve interval observational studies that track circulating tumour DNA (ctDNA). However, sample collection for such studies are not priorities and involve patients in follow-up; their hospital appointments have been cancelled in recent months. Moreover, even before the COVID-19 pandemic access to clinical trials could be patchy and lacking in patient diversity in places across and beyond the UK ( Kerr & Cunningham-Burley, 2015). During the initial months of the pandemic clinical trial recruitment ground to a halt in the UK which meant for many patients a missed window of opportunity to access potentially life-extending drugs.

The current challenge of conducting social science research in the clinical settings means examining the broader social impact of the pandemic on the practices of personalisation are yet to fully materialise, teasing out how the pandemic intensifies micro and macro-level asymmetries of personalised cancer care is particularly important. As patients navigate an even more uncertain landscape of complex care, the pandemic reminds us of the ongoing, and in fact urgent need to scrutinize the meaning of personalisation. Indeed, the virus continues to demonstrate how a truly personalised approach in healthcare should not be confined to the biological aspects of treatments but must also consider the needs of the patient from a holistic point of view ( Day *et al.*, 2016; Kerr *et al.*, 2021; Prainsack, 2018).

## A third “C”: The need for critique

While seeking to acknowledge the impact of the pandemic on research and care infrastructures around cancer, we invite caution towards totalising tendencies. The risk of covidizing cancer resembles the #ForgottenC, an online hashtag becoming popular in online platforms among charities and other advocacy groups. Remembering cancer during the pandemic presumes to know and thus recall cancer as a figure being forgotten. We want to call attention to work carried out in clinical and non-clinical contexts that highlights the diverse and unequal resources afforded to people using health services in the UK. We encourage our colleagues to look at cancer as a biosocial phenomenon rather than limiting it to the simplified narrative portrayed as part of charitable fundraising efforts.

Reckoning with a #ForgottenC is to do critical work with longer histories of managerialism, streamlining and efficiency saving in the context of public sector austerity, privatisation, biotech and pharma profiteering, and the degradation of hospital estates. How we remember one C should put to work another: “critique”. Critical work involves considerations of our own privileges, biases and limitations, especially our dependencies on the private wealth of charitable organisations, conditions what social science can do within a pandemic. It involves, again, reckoning with our dependencies, antagonisms, and entanglements with biomedicine. Building on our expertise as social science scholars in the field of cancer, we sought to outline what a critical, reflexive and provisional approach to Cancer and COVID-19 may involve.

## Data availability

### Underlying data

No data are associated with this article.
