# Supplementary material for: Cancer, COVID-19, and the need for critique
Source: Wellcome Open Res. 2021 Jun 11;5:280. Originally published 2020 Nov 27. [Version 2] doi: 10.12688/wellcomeopenres.16404.2 (PMC7839273; doi:10.12688/wellcomeopenres.16404.2)
Supplement: Peer review history

# Peer review: Reviewer response for version 1

**DOI:** 10.21956/wellcomeopenres.18047.r41896

**Published:** 2021-01-26

**Authors:** Christine Schmid

**Affiliations:** 1 Department of Psychology and Ergonomics, Technical University of Berlin, Berlin, Germany

I enjoyed reading the open letter “Cancer, Covid-19, and the need for critique”. It is a very well-written piece contributing to discussions about the entanglements between Covid-19, cancer care and social science research. The open letter addresses the impact of the pandemic on cancer care, on patient's lives and points out that this current “moment of crisis” reveals a fragmentation in cancer care that already existed before.

 However, the main argument, as I understand it, is a call for the importance of - underestimated and neglected within the field cancer research in times of the pandemic - qualitative social science research.

 Overall, I think this argument, and in particular its development from the concrete example of the fragmentation of cancer care to the need for critical social scientific debate, works.

 However, it might also be this structure that leaves me as a reader missing clarity as to what the authors' main intention or main argument is.

 For instance, the description of fragmentation could be elaborated on in more detail, if this is an example for why and how social scientist can contribute to critical research on cancer care. Otherwise, in the end, it is still unclear how this exactly becomes relevant or critical in practice. At the same time, I was wondering, if this reasoning for social sciences as a primarily critical form of knowledge production - without elaborating on its contributions in more detail - reinforces an image of a distanced “critique from outside”. Following Latour (2014) this form of deconstructive critique from social sciences is not effective anymore in order to contribute to current social questions. But this might be a follow-up question and not so vital to the letter.

 By the same token, I was wondering why the authors did not refer to some more of their own work in order to clarify abovementioned fragmentation – this would additionally support the argument that research should not be divided into pre- and post- COVID-19 research.

 Also, the examples in the open letter are based on a specific national cancer care context. For me, as a non-UK researcher, it would help to know more about the division into public and private health care to understand the argument.

 On the other hand, I feel that the part on research practices during COVID-19 beyond the description of the current difficulties is too general and only loosely tied to the other parts. I think it could gain if it would be tied to either concrete options and examples of how the authors currently work, or to the question of fragmentation (e.g. along the lines of: does the use of digital counselling in cancer care versus personal counselling lead to another variant of fragmentation?).

 Furthermore, I would like to know more about the context of the workshop that led to this open letter. Why did you meet up and organize a workshop? What was your initial question? And how did you end up with this open letter?

 All these comments can be addressed relatively easy and I hope that they help to strengthen and clarify the important argument in this text.

Does the article adequately reference differing views and opinions?

Yes

Are all factual statements correct, and are statements and arguments made adequately supported by citations?

Yes

Is the Open Letter written in accessible language?

Yes

Where applicable, are recommendations and next steps explained clearly for others to follow?

Not applicable

Is the rationale for the Open Letter provided in sufficient detail?

Yes

Reviewer Expertise:

Medical Anthropology, Social Anthropology of Science and Technology, Anthropology of Experience, Hospital Ethnography.

I confirm that I have read this submission and believe that I have an appropriate level of expertise to confirm that it is of an acceptable scientific standard.

# Response

**Published:** 2021-06-03

**Authors:** Cinzia Greco

**Affiliations:** University of Manchester, UK

We want to thank Caitjan Gainty and Christine Schmidt for the positive evaluation of our open letter and their constructive comments. We have revised the open letter in several passages to address some of their interventions – notably, on issues related to the contribution of qualitative social science, the meanings of “covidization”, and the organisation of healthcare in the UK. At the same time, we aimed to keep the open letter relatively brief, reflecting on some of the comments in more detail below.

 We have added a few details in the letter itself explaining the context and purpose of our workshop. Having met each other in previous academic meetings, we saw the workshop as an opportunity to discuss, informally, how new difficulties hindered our fieldwork research in the UK in the context of the pandemic. Rather than guiding our reflections with the aid of a set of specific questions, the analysis we presented in the open letter emerged from loosely structured exchanges that took place throughout the process. Cinzia Greco and Ignacia Arteaga collated the reflections presented by participants and started further conversations with all the authors around the text that became the current open letter. The issues we decided to touch upon, expressly limited, aimed to represent some of the domains we were exploring at the moment of writing.

 Many thanks to Caitjan Gainty for the remarks about cancer not being exclusively defined as a clinical reality. The same conceptual standpoint is seminal in our work. We agree that cancer is no different from other conditions, even though its high prevalence and public imagery continually shape healthcare dynamics and patients’ relationships. Consequently, we agree that the issues we describe are not limited to cancer. Our choice to focus on cancer is linked to the fact that we have specific expertise as a group of researchers and because specific concerns have been raised regarding the on-going disruption to oncology services caused by Covid-19 within clinical, patient and charity communities (e.g. CRUK). We believe that a more extensive discussion of the impact of the pandemic on healthcare, in general, would need different data-gathering and analysis techniques and a format different from an open letter.

 In response to both reviewers’ remarks about our disciplinary contribution to understanding the pandemic and attending to some of its consequences, we have also included a paragraph outlining some of the ways in which social science contributes to the understanding of the pandemic. On the one hand, we can conceptualise the pandemic as a “social fact” that must be understood and unpacked in its differential effects in various domains. On the other hand, the pandemic is also conceived as a reality to be intervened upon to aid the design of recovery plans informed by the relevant issues in people’s social worlds. Importantly, changing the vantage point includes examining and verbalising the normative assumptions that inform policy decisions which is in itself a mode of political intervention.

 We have now revised the passage describing the critique around the “covidization” of research, following and expanding on Pai’s argument. We underline that our use of the concept refers specifically to problematic aspects of the interpretation of the pandemic as a cultural form. These include issues in the larger organisation of scientific research. These epistemological issues have to do with the reproduction of long-standing hierarchies of evidence and negative ways in which the COVID pandemic might transform medical practices in a post-pandemic context by diverting resources and attention from long-standing issues that also merit scholarly attention.

 Our worries, shared with Pai, are that part of the exceptional effort in terms of research, dissemination, and funding directed at the study of COVID has the potential to overlook previous social and medical issues. In this line, COVID exacerbates and highlights questions for us, rather than creating them ex-novo. We see the problem as partly due to a lack of balance in the research produced. More than this, it is the risk of doing research that disconnects the impact of the pandemic from longstanding issues in healthcare.

 We are also concerned that this strategic move might produce a limited understanding of the pandemic by ignoring pre-existing problems that were exacerbated rather than produced by the pandemic. We are among the many who hope the pandemic should be an opportunity to correct part of what was wrong in healthcare. Yet, we see the danger that the post-pandemic might also result in the roll-back of treatments and services that, although convenient for the financial budget of healthcare trusts, is detrimental to patients. Managers’ realisation that their hospital trusts and general practices can make efficiencies after the pandemic risks healthcare becoming even more unequal and fragmented.

 In a context where some community groups have already troubled stories in their interaction with healthcare, we risk further alienating and excluding those who do not have the resources to become “activated” in their communities, cannot “connect” via virtual technologies, or cannot afford new forms of “patient work” that are now required. Here, “covidization” does not refer to the main topic we are discussing in the letter. Instead, it refers to a specific problem to avoid: addressing COVID by ignoring its links with pre-existing situations and exclusions in healthcare, thereby aggravating them.

 For the readers unfamiliar with the British context, we have added a brief passage on the NHS’s relation to the public, the market, and the state. The NHS has historically stood out in contrast to the role of the private social and private for-profit actors elsewhere in Europe and, more so, in North America. However, it has seen a steady increase of private actors since the 1980s. While the UK is still a nation with a prominent role given to the state in terms of healthcare funding, we see the continuous outsourcing of several in-house services to private companies, including for-profit entities. More relevant for our analysis, private non-profit charities in the UK have an essential role in funding translational and population research, additional clinical services, and professional positions, such as nursing and palliative care.

 We thank the reviewers for their questions and suggestions again. We hope that this response illuminates some of the issues that – no doubt – we need to continue reflecting upon.

 Yours,

 Cinzia Greco, Ignacia Arteaga, Clara Fabian-Therond, Henry Llewellyn, Julia Swallow and William Viney.

# Peer review: Reviewer response for version 1

**DOI:** 10.21956/wellcomeopenres.18047.r41686

**Published:** 2020-12-15

**Authors:** Caitjan Gainty

**Affiliations:** 1 Department of History, King's College London, London, UK

This open letter is rich and thoughtful. It offers, for one thing, a review of the ways in which the cancer care industry and its actors all along the line - including its frontline physicians and patients – have been impacted by the COVID-19 pandemic. Here, the letter points to accounts of patients whose treatments have been disrupted and whose status within healthcare has changed: from those heavily dependent on the system to those too ill to be treated should they contract severe COVID.

 At the same time, the letter points out the dangerous potential for attribution errors during a pandemic, noting that the so-called “covidization” of cancer cuts two ways: in its implication that COVID-19 has actively disrupted cancer research and therapy, it notes the novelty of a once-in-a-generation health event on all aspects of the “regular” system of the cancer care industry . But it also introduces the risk that too much attention to this specialness will paper over what are actually longer-standing continuities, forces or factors attributable to causes outside of COVID-19.

 But both of these themes are subordinate to what I take to be the letter’s main argument: that those who study cancer from the perspective of the social studies of science, technology and medicine ought to be considered critical players in this discussion – as critical, in fact, as any of the other knowledge-making actors in cancer care – because the factors that they are uniquely equipped to deal with, the biosocial, cultural, economic, political, etc, are just as fundamental to the creation of cancer as any other. In this, the authors are calling for recognition that disease is not just a clinical creation and that as a result the factors relevant to understanding, signifying and managing a disease cannot be limited only to those who experience it clinically.

 The authors underscore this point in a variety of useful ways, and I think the argument works by and large, though it remains a question to me whether this is something that the authors feel is specific only to cancer or whether there discussion here is actually more radical, that when we acknowledge, as we publicly do, that medicine and health are more than just clinical entities, it makes little sense to prioritise clinical entities as those suited to define its significance.

 Given how potentially important the authors indicate the role of the social scientist is, it feels a bit of a let down that they do not indicate more precisely what it is that the presence of social scientists in cancer’s inner knowledge-making circle might yield. The language they use to describe their role is remarkable for its passivity. Reseachers “bear witness to” or are “enable[ed]” to “document and better understand”. This leaves a hole at the veritable center of the argument: what is it, besides watching and documenting, that social scientists are able to contribute? The translation from arguing the case from a matter of academic necessity to arguing it as a matter of real-time, real world necessity is not fully described here, even though the real world significance of the authors’ work cries out for acknowledgment. What would change if social scientists were in the mix?

 I wonder if perhaps this omission is a translational one: the difficulty of describing one’s significance in terms other than those internal to one’s own work is well known. And if so, I wonder whether the letter is not otherwise troubled by this translation, or perhaps by the artefactual remains of what were clearly wide-ranging and dynamic conversations had at the conference in September. Among other things, the discussion of the difficulties of social scientific research in a pandemic feels very specific, yet the letter is not necessarily otherwise specifically about the difficulties particularly attendant with social science research in a pandemic exactly. The situation is similar with the discussion of the NIHR: a very specific reference that feels out of keeping with some of the more thematic points made in the letter, even if it used to make a point about research priorities. And the discussion of personalized medicine, while interesting and thoughtful, also seems awkwardly bent toward the matter at hand. The reference to Steve Sturdy’s work here, indeed, feels a bit of a red herring: I read that work as a rich provocation about how to (re)signify personalized medicine. But this section doesn’t follow through on Sturdy’s notions of bioetch’s “promissory economy” and instead almost seems to double down on a slightly more facile notion of personalized medicine that, given the theoretical richness of their account in other places, seems just out of place.

 Both of the above can be addressed relatively easily and are not so vital to the letter's main argument. What should be addressed, I think, is the uncertainty attached to the notion of covidization. Initially, covidization seems primed to refer in quite a facile way to research costs. The referenced text focuses on researchers attracted to study COVID whose loss aggrieves the community of researchers they’ve left; whose help, for lack of expertise, is actually not helpful to those they’ve joined; and who thus add to a cacophony of scientific voices rather than becoming a part of a single cohesive sensibility about how to think about and what to do about COVID. This rather reductive view (of covidization but also of scientific research, for that matter) feels out of place, and the examples the authors include of the covidization of cancer don’t clarify how their meaning of the term relates to this one.

 It seems to me that covidization for the authors ultimately seems to signify something to do with an inability to strike a balance between the novelty of the pandemic, at least in our lifetimes, and its non-novelty, both as a historical phenomenon and as a prism onto healthcare in real time. Does COVID represent a change in kind or a change in degree?

 If this reading is right, then we are left with some uncertainty about the status of the letter. Is it suggesting something about the attendant and specific difficulties of cancer and COVID, of cancer as a covidized disease, and therefore the need for social scientific contributions? If so, does this contradict the richness of the definition of covidization that the authors provide? Or are the authors saying that now, as at all times, social scientists need to be involved in the study of cancer? And if so, does this mean that their intonation of COVID here is too strong? Some resolution to this conceptual difficulty would be in order. If, for example, it is that COVID has exacerbated and/or laid bare for us what is already true about cancer, then this is certainly worth saying. Whatever their position, greater clarity would help a great deal to bring out the really important message that this letter contains.

Does the article adequately reference differing views and opinions?

Yes

Are all factual statements correct, and are statements and arguments made adequately supported by citations?

Yes

Is the Open Letter written in accessible language?

Partly

Where applicable, are recommendations and next steps explained clearly for others to follow?

Not applicable

Is the rationale for the Open Letter provided in sufficient detail?

Yes

Reviewer Expertise:

History of health and healthcare.

I confirm that I have read this submission and believe that I have an appropriate level of expertise to confirm that it is of an acceptable scientific standard.
